# Amygdala size varies with stress perception

**DOI:** 10.1016/j.ynstr.2021.100334

**Published:** 2021-05-01

**Authors:** Inês Caetano, Liliana Amorim, José Miguel Soares, Sónia Ferreira, Ana Coelho, Joana Reis, Nadine Correia Santos, Pedro Silva Moreira, Paulo Marques, Ricardo Magalhães, Madalena Esteves, Maria Picó-Pérez, Nuno Sousa

**Affiliations:** a Life and Health Sciences Research Institute (ICVS), School of Medicine, University of Minho, 4710-057, Braga, Portugal; b ICVS/3B's, PT Government Associate Laboratory, 4710-057, Braga/Guimarães, Portugal; c Clinical Academic Center – Braga, Braga, Portugal, 4710-057, Braga/Guimarães, Portugal; d Association P5 Digital Medical Center (ACMP5), 4710-057, Braga, Portugal; e NeuroSpin, Institut des Sciences du Vivant Frédéric Joliot, Commisariat à l’Énergie Atomique et aux Énergies Alternatives, 91191, Gif-Sur-Yvette, France; f Université Paris-Saclay, 91191, Gif-Sur-Yvette, France

**Keywords:** Perceived stress, Amygdala, Voxel-based morphometry, FreeSurfer, Healthy subjects, PSS10, 10-items Perceived Stress Scale, FSL, FMRIB Software Library, VBM, Voxel-based morphometry, ROI, Region-of-interest, TFCE, Threshold-free cluster enhancement, FWE-R, Family-wise error rate, GM, Gray matter, WM, White matter, eTIV, Estimated total intracranial volume, M, Mean, SD, Standard deviation

## Abstract

Stress is inevitably linked to life. It has many and complex facets. Notably, perception of stressful stimuli is an important factor when mounting stress responses and measuring its impact. Indeed, moved by the increasing number of stress-triggered pathologies, several groups drew on advanced neuroimaging techniques to explore stress effects on the brain. From that, several regions and circuits have been linked to stress, and a comprehensive integration of the distinct findings applied to common individuals is being pursued, but with conflicting results. Herein, we performed a volumetric regression analysis using participants’ perceived stress as a variable of interest. Data shows that increased levels of perceived stress positively associate with the right amygdala and anterior hippocampal volumes.

## Highlights

• Discrepant stress effects on morphology are reported in the literature.
• Stress definition and analysis software are the main causes of conflicting findings.
• Psychological measures and multi-technique analysis are highly recommended.
• We found that perceived stress positively associates with right amygdala volumes.

## 1 Introduction

When facing a stressor, and depending on its type, duration, and individual vulnerability, a subject triggers a variable response that can be partially measured; however, it is important to note that, largely, stress is a subjective perception (Godoy et al., 2018; Novais et al., 2017). Indeed, stress, and the individual perception of stress, is a key factor in mental health. Either by the time pressure of a busy life, economic factors, professional questions (Everaerd et al., 2020), personal and social relationships, or individual susceptibility (Duman et al., 2016), stress has invaded our lives to become a common presence in modern society (Kalisch et al., 2017; Lucassen et al., 2014). In line with the increasing number of people with stress symptoms, several studies have shown an association between stress and neuropsychiatric conditions such as major depression disease (Hammen, 2005; Kendler et al., 1999; Melchior et al., 2007; Welberg, 2014), anxiety (Melchior et al., 2007), schizophrenia (Walker et al., 2008), bipolar (Kim et al., 2007) and posttraumatic stress disorder (Brewin et al., 2000). As a result, there has been a significant effort of the scientific community to build a map of brain regions impacted by stress (Sousa, 2016) as well as their functional consequences both in physiological and pathological conditions (Lucassen et al., 2014; Novais et al., 2017). Such effort has resulted in an impressive collection of data, but unfortunately with several discrepancies. In terms of cortical volumetry, some studies showed reduced volumes of the prefrontal cortex (PFC), orbitofrontal cortex (OFC), anterior cingulate cortex (ACC), superior temporal gyrus (STG) and insula (Ansell et al., 2012; Moreno et al., 2017; Savic et al., 2018), whereas others failed to find such volumetric differences in the same cortical regions or even found potential signs of the opposite (Merz et al., 2019; Piccolo and Noble, 2018; Soares et al., 2012; Zhang et al., 2018). Interestingly, two opposite trends on the structure of subcortical regions implicated in the stress response are extensively reported in the literature and need to be revised. While some studies show, for example, volumetric increases in several subcortical brain regions, in particular the amygdala, (De Bellis et al., 2000; Henigsberg et al., 2019; Klaming et al., 2019; Kuo et al., 2012; Lucassen et al., 2014; Morey et al., 2016; Novais et al., 2017; Pinto et al., 2015; Schienle et al., 2011), others fail to reproduce these findings (Herringa et al., 2012; Karl et al., 2006; Kitayama et al., 2005; Koshiyama et al., 2018; Magalhães et al., 2018; Morey et al., 2012; Soares et al., 2012; Vriend et al., 2016; Zimmerman et al., 2016).

A significant part of such conflicting findings derives from methodological approaches. On one hand, there are technical issues related to image processing. In fact, particular attention to preprocessing and analysis steps is required when conducting neuroimaging studies. For instance, Katuwal et al. demonstrated that brain volume estimates were dependent on the software chosen (SPM, FSL, and FreeSurfer), leading to differences upon between-group comparisons, with some results presenting opposite directions (Katuwal et al., 2016). Similarly, Grimm et al. have shown large differences upon amygdala and hippocampal volumes that were computed through manual segmentation, using FreeSurfer, and using VBM (implemented in SPM8), highlighting the disparities across methods (Grimm et al., 2015). On the other hand, there are important issues in study design. In fact, stress is not a homogenous concept and group comparisons always suffer from the intra-group variability in measurements (namely in endocrine measurements). Interestingly, the 10-items Perceived Stress Scale instrument (PSS10) has been well validated, both for healthy and pathological populations (Fliege et al., 2005; Trigo et al., 2010), with several studies showing its psychometric qualities on the individual quantification of perceived stress levels (Lee, 2012; Soares et al., 2012). Thus, taking into account that stress perception is a central element in the present study, we opted to use the PSS10.

Herein, we have tackled such methodological issues by performing a study that explores the association of perceived stress scores using the PSS10 questionnaire and the volumes of subcortical brain regions determined with multiple techniques. By doing so, we avoid the bias of group classification and high variability between individuals in cortisol measurements, but also the bias of distinct software/pipelines for volumetric analysis.

## 2 Material and methods

### 2.1 Ethics statement

The present study was conducted in accordance with the principles expressed in the Declaration of Helsinki (59th amendment) and approved by the national and local ethics review board committees (*Comissão Nacional de Protecção de Dados*, C*omissão de Ética para a Saúde of Hospital de Braga*, and *Subcomissão de ética para as ciências da vida e da saúde* from University of Minho). The study aims were explained to all participants and all signed informed consent.

### 2.2 Participants and study design

The present study gathered 50 participants recruited at the School of Medicine, University of Minho, and at Faculty of Medicine, University of Porto. Primary exclusion criteria included inability to understand the informed consent or its non-acceptance, individual choice to withdraw from the study, incapacity and/or inability to attend the MRI session and/or diagnosed neuropsychiatric disorder or any other comorbidity of the central nervous system.

Structural acquisitions from all participants were collected as well as the PSS10 questionnaire. This scale reports to the previous month and is a reliable and validated self-administered instrument largely used to assess chronic psychosocial stress both in clinical and healthy adult samples (Cohen et al., 1983; Fliege et al., 2005; Liston et al., 2009; Soares et al., 2012; Trigo et al., 2010). Additionally, before the MRI acquisition, half of the participants collected saliva samples for posterior analysis of cortisol, the primary stress hormone.

To unveil associations between morphometry and psychological stress, a volumetric regression analysis with PSS10 scores as a variable of interest was conducted, using the FSL-VBM software (voxel-based method). As a complementary analysis, we also addressed our question using FreeSurfer (ROI-based analysis).

To further explore the association between cortisol and morphometry, we performed an additional volumetric regression using FreeSurfer software.

### 2.3 Participants characterization and cortisol measurement

The demographic, psychological and endocrine characterization of participants was made using SPSS version 23 (IBM, SPSS, Chicago, IL, USA). The normality assumption for each variable was tested and non-parametric tests used when the assumption not met.

Due to the use of age and sex as covariates in all MRI analyses, a correlation between PSS10 scores/cortisol and age/sex was performed to disclose the existence of a possible association that could affect volumetric regression results.

For cortisol measurement, saliva samples were collected in the morning, between 9 a.m. and 12 a.m., using Salivette collection devices (Sarstedt, Germany). The samples were stored at −22 °C until the biologically active, free fraction of cortisol be analysed with an immunoassay (IBL, Hamburg).

### 2.4 MRI data acquisition

Imaging sessions were conducted at Hospital of Braga (Braga, Portugal) on a clinical approved Siemens Magnetom Avanto 1.5 T MRI scanner (Siemens Medical Solutions, Erlangen, Germany), using a 12-channel receive-only head coil. The imaging protocol consisted of a T1 high-resolution anatomical sequence. The established clinical protocols for the 3D magnetization prepared rapid gradient echo (MPRAGE) were performed with a repetition time (TR) = 2.4/2.7 s, echo time (TE) = 3.62/2.73 ms, 160/176 sagittal slices with no gap, field-of-view (FoV) = 234/256 mm, flip angle (FA) = 7/8°, in-plane resolution = 1.0/1.2 × 1.0/1.2 mm^2^ and slice thickness = 1.0/1.2 mm.

### 2.5 MRI data preprocessing

Before any data processing, a certified neuro-radiologist visually inspected all acquisitions and confirmed that they were not affected by critical head motion and that the participants had no brain lesions or pathologies. Preprocessing was made using fMRIPrep version 1.4.1 (Esteban et al., 2019) (RRID:SCR_016216), which is based on Nipype 1.2.0 (Gorgolewski et al., 2011, 2017) (RRID:SCR_002502).

Each anatomical T1-weighted (T1w) image was corrected for intensity non-uniformity (INU) with N4BiasFieldCorrection (Tustison et al., 2010), distributed with ANTs 2.2.0 (Avants et al., 2008) (RRID:SCR_004757), and used as T1w-reference throughout the workflow. Then, the T1w-reference was skull-stripped with a Nipype implementation of the *antsBrainExtraction.sh* workflow (from ANTs), using OASIS30ANTs as target template and segmented into cerebrospinal fluid (CSF), white matter (WM), and gray matter (GM) using *fast* (Zhang et al., 2001) (FSL 5.0.9, RRID:SCR_002823). The reconstruction of brain surfaces was made with *recon-all* (Dale et al., 1999) (FreeSurfer 6.0.1, RRID:SCR_001847), and the previously brain mask estimated was refined with a custom variation of the method to reconcile ANTs-derived and FreeSurfer-derived segmentations of the cortical GM of Mindboggle (Klein et al., 2017) (RRID:SCR_002438). The images were non-linearly transformed into standard space ICBM 152 Nonlinear Asymmetrical template (Fonov et al., 2009) (version 2009c; RRID:SCR_008796; TemplateFlow ID: MNI152NLin2009cAsym) with *antsRegistration* (ANTs 2.2.0), using brain-extracted versions of both T1w reference and T1w template.

### 2.6 FSL-VBM analysis

The voxel-based morphometry analysis was performed using FSL-VBM (Douaud et al., 2007) (http://fsl.fmrib.ox.ac.uk/fsl/fslwiki/FSLVBM) which is an optimized VBM protocol (Good et al., 2001) implemented through tools from the FMRIB Software Library (Smith et al., 2004) (FSL 5.0.9, www.fmrib.ox.ac.uk/fsl). The recommended analytical pipeline was followed, however, image skull-striping, segmentation into tissue classes, and non-linearly transformation to standard space steps were not performed since they had already been computed during preprocessing. To create a left-right symmetric study-specific gray matter template, the standardized GM images obtained with the fMRIPrep were averaged and flipped along the x-axis. In order to build an unbiased template, an equal number of images acquired with each configuration was used (32 in total). At this stage, all native GM images were non-linearly registered to the study-specific template created and “modulated” to correct for local expansion and/or contraction due to the non-linear component of the spatial transformation. The Jacobian modulation does not include the affine part of the registration meaning that the images are already normalized for total cranial volume differences (corrections upon total cranial volumes are only required when modulation includes the affine part (Good et al., 2002)). Then, the modulated GM images were smoothed using an isotropic Gaussian kernel with a sigma = 3 mm (corresponding to FWHM = 7 mm). Finally, considering PSS10 scores as a variable of interest and sex, age, and MRI configuration, as between-subject covariates, a voxelwise General Linear Model (GLM) was applied using non-parametric permutation-based testing (5000 permutations), with TFCE-based thresholding, upon a subcortical mask created according to FreeSurfer subcortical automatic segmentation (Fischl et al., 2002) (bilateral regions of the thalamus, caudate, putamen, pallidum, hippocampus, amygdala, and accumbens area). Correction for multiple comparisons across space were performed with a significance of 0.05, and, after visual inspection, statistically significant clusters obtained were reported also according to FreeSurfer labeling. The voxels with higher probability in each cluster were defined as peaks and the averaged probability over all the voxels in the cluster was considered for global cluster statistics.

### 2.7 FreeSurfer ROI-based analysis

The morphometry ROI-based analysis was conducted for each region individually. Firstly, subcortical volumes of participants were computed, and then, the statistical analysis upon those volumes was individually conducted.

For volumes computation, the FreeSurfer derivatives of the fMRIPrep were used. The general FreeSurfer (Fischl, 2012) (http://surfer.nmr.mgh.harvard.edu) pipeline implements 31 processing steps, including motion correction, spatial normalization to Talairach standard space, intensity normalization, skull stripping, and segmentation of WM, cortical, and subcortical regions. In the fMRIPrep, the processing steps of the FreeSurfer pipeline are aggregated in 3 phases. Firstly, a subject T1w structural image is initialized and a basic reconstruction, excepting skull-stripping, is performed using the *autorecon1* (first 5 preprocessing steps of *recon-all* (Dale et al., 1999) function, excluding step 5, the skull-stripping)*.* Secondly, a brain mask that was previously computed in the fMRIPrep workflow (using *antsBrainExtraction.sh*) is directly injected into the appropriate FreeSurfer location, in place of the skull-stripping step that was not performed before. Herein, this external brain mask is also refined using the internal mask of the FreeSurfer's *aseg.mgz* segmentation, reconciling ANTs-derived, and FreeSurfer-derived segmentation of the cortical GM of Mindboggle (Klein et al., 2017). Finally, the third phase resumes *recon-all* execution (*autorecon2* and *autorecon3*), dividing all the remaining FreeSurfer steps into sub-stages, to use resources more efficiently. For the main purpose of this work only volumes resulting from the automatic subcortical segmentation (Fischl et al., 2002) were used (bilateral thalamus, caudate, putamen, pallidum, hippocampus, amygdala, and accumbens area). To best replicate the FSL-VBM analysis, all the volumes were corrected for individual GM, and multiplied by 100 to avoid a large number of decimal digits.

For statistical analysis, multilinear regression models with each ROI volume as the dependent variable and PSS10 scores (or cortisol), age and sex as independent variables were established. The models were computed in MATLAB and the Bonferroni-Holm (1979) correction for 14 multiple comparisons were used to calculate the corrected *p*-values. When two independent terms were statistically significant in the same model, the interaction effect between those terms was explored by including the interaction factor on the model. Additional analyses on the anterior (head) and posterior (body and tail) hippocampus were conducted to explore the structural segregation of this region. For hippocampal segmentation, the FreeSurfer version 7.1.1 was used (Iglesias et al., 2015).

Linear regressions and independent-samples *t*-test were used to further explore the statistical significance of independent terms of the multilinear models obtained.

As exploratory analysis, the regression between cortical brain volumes and PSS was also conducted.

Finally, an independent sample *t*-test regarding the estimated total intracranial volume (eTIV), and a Mann-Whitney *U* test regarding GW, were made to confirm that no morphometry differences are caused due to the slight protocol disparities.

## 3 Results

### 3.1 Cohort characterization

The demographic and psychological characterization of participants, as cortisol measurements, are presented in Table 1. No correlation was found between PSS10 scores and age/sex (*r* (50) = 0.091/0.077, *p* = 0.530/0.716), nor between cortisol measurements and age/sex (*r* (25) = −0.055/0.242, *p* = 0.789/0.243). Taking into consideration the usage of age and sex as covariates in MRI analysis, the absence of a significant correlation ensures that the effects of stress in the regression are not decreased or affected by the usage of covariate variables.

**Table 1:** Demographic, psychological and endocrine characterization of participants.

	*N*	Mean (±SD)
DEMOGRAPHIC		
Age, years	50	24.30 ± 1.81
Male	15 (30%)	
Female	35 (70%)	
**PSYCHOLOGICAL**		
PSS10 scores	50	26.2 ± 7.14
**ENDOCRINE**		
Cortisol	25	0.32 ± 0.21
Male	7 (28%)	
Female	18 (72%)	

Regarding the association between stress measurements, no correlation was found between PSS10 scores and cortisol levels (*r* (25) = 0.107, *p* = 0.612).

Descriptive statistics of subcortical volumes, GM, WM, and estimated total intracranial volume (eTIV) obtained through FreeSurfer are presented in supplementary Table A.1**.** When comparing results from both acquisition protocols, no differences were found in subjects' eTIV (*t* (48) = 1.915, *p* = 0.061), nor in subjects’ GM (*U* = 359, *p* = 0.070).

### 3.2 Volumetric regression with PSS10

**Fig. 1:**
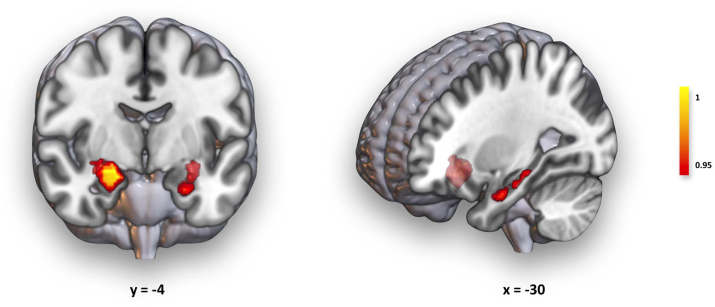
Results from volumetric regression with PSS10 evaluated through FSL-VBM. A positive statistically significant association between PSS10 scores and two main subcortical clusters is observed. On the left, the biggest cluster is mainly composed of the right amygdala (peak), embracing part of the right hippocampus and a small portion of the right putamen. On the right, a smaller cluster is observed the in left hippocampus (peak) and left amygdala. Following the FSL-VBM pipeline, after brain extraction and segmentation, a study-specific GM template was created, upon which all GM images were registered. During this registration step, a compensation for the non-linear component of the transformation is introduced by the FSL-VBM protocol, which already adjusts for intracranial differences. Therefore, only age, sex, and MRI parameters were defined as covariates, and TFCE and FWE-R correction at a significance level *α* = 0.05 were used.

As shown in Fig. 1 and Table 2, when exploring the association between PSS10 and morphology through FSL-VBM, a positive statistically significant association within 3 clusters is observed. The largest, and the most significant, cluster is centered in the right amygdala (peak), including also part of the right anterior hippocampus and a small portion of the right putamen. In the left hemisphere, a more dispersed cluster essentially extends through the hippocampus (peak), also comprising a portion of the amygdala, was found. Finally, data reveals a very small cluster, composed of 3 voxels of right pallidum. In all cases, these brain regions displayed higher volumes in subjects with higher stress perception.

**Table 2:** Results from the volumetric regression analysis with PSS10 evaluated through VBM and FreeSurfer. Positive statistically significant association within PSS10 scores and subcortical regions are observed for both methods. On the left, clusters resulted from the volumetric regression analysis with perceived stress scores on FSL-VBM and respective statistics. In the middle, brain region classification according to FreeSurfer subcortical segmentation (*aseg.stats*). On the right, significant results of FreeSurfer analysis, with all regions identified in VBM-FSL clusters represented independently of its significance, plus all regions with significance on FreeSurfer before correction for multiple comparisons.

FSL-VBM								Brain Region	FreeSurfer		
Cluster index	Cluster size	Peak					*n* voxels FSL-VBM cluster		*n* voxels FreeSurfer ROI	ROI	
		*t*-value	*p*(FWE-corr)	coordinates (*mm*)						*p*-value	*p*(Bonf-corr)
				***x***	***y***	***z***					
1	310	4.39	0.007	20	−4	−12	157	54_R Amygdala	263	**<0.001**	**<0.001**
							138	53_R Hippocampus	658	**0.021**	0.248
							15	51_R Putamen	831	0.756	2.520
2	116	3.95	0.029	−30	−22	−20	99	17_L Hippocampus	634	0.093	0.838
							17	18_L Amygdala	219	**0.013**	0.163
3	3	3.15	0.049	20	−4	−6	3	52_R Pallidum	207	**0.025**	0.271
							–	58_R Accumbens-area	83	**0.033**	0.326

TFCE and FWE-R correction at a significance level of 0.05 for FSL-VBM analysis; Multilinear regression with Bonferroni-Holm correction for 14 comparisons in FreeSurfer analysis. ROI. Region of interest; R. right; L. left.

**Fig. 2:**
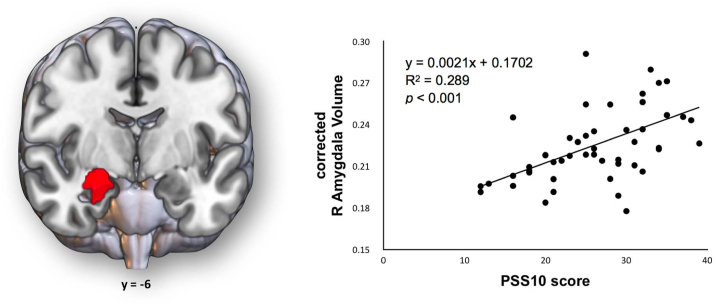
Results from volumetric regression with PSS10 evaluated through FreeSurfer. A positive statistically significant association between PSS10 scores and right amygdala was observed, after correction for multiple comparisons. On the left, a representation of the *54_R Amygdala* cluster from FreeSurfer subcortical regions labelling. On the right, a graphical representation of the model with significance between PSS10 scores and right amygdala volumes corrected for GM, and age/sex covariates; the equation represents the correlation between the PSS10 scores and corrected right amygdala volumes, and not the global model *per se*. Brain volumes were computed using FreeSurfer subcortical output and corrected for individual GM to best replicate the VBM regression analysis. Multilinear regression models with ROI volumes as dependent variables and PSS10 scores, age, and sex as independent variables were established. The models were computed using the *regstats* function in MATLAB and the Bonferroni-Holm correction for 14 multiple comparisons was used to calculate the corrected p-values. Statistical significance was established for *α* = 0.05.

Results from volumetric regression with perceived stress evaluated through FreeSurfer also show a statistically significant positive association between PSS10 scores and the volume of the right amygdala. In Fig. 2, a representation of such association between stress perception and the size of the right amygdala is displayed, as well as the graphical representation of the significant model obtained, corrected for age and sex covariates. The positive associations between PSS10 and right pallidum, right hippocampus, left amygdala and right accumbens area were noted, but did not survive to multiple comparison correction (See Table 2 for more information on these regions, and supplementary Table A.2 for a detailed description of all models obtained).

When exploring the association between PSS scores and hippocampal segregation, a statistically significant association was observed only in the anterior hippocampus (see Supplementary Table A.3 for details).

The exploratory analysis across the cortical regions, revealed no statistically significant results.

### 3.3 Volumetric regression with cortisol

A multilinear model revealed a significant negative association between cortisol measurements and the left thalamus volumes. Moreover, a positive association with sex and left thalamus was also observed, which due to the covariate codification (females as 0 and males as 1), indicates that being a male positively contribute to having a bigger volume of left thalamus, and, in contrast, being a female contribute to having smaller left thalamus volume. (See supplementary Table A.4 for a description of all models obtained). When including in the model the interaction factor between cortisol and sex, no statistical significance for the interaction was observed (*p* = 0.3782), which indicates that there are no differences in the association of left thalamus volumes and cortisol levels between males and females. Further between-group analysis upon sex indicates that left thalamus volumes are higher in males (*M* = 1.23 ± 0.082) than in females (*M* = 1.15 ± 0.067), when controlling for cortisol levels and age (*t* (23) = 2.225, *p* = 0.034, *d* = 0.94). In Fig. A.1, graphical representations of the left thalamus model, considering cortisol/sex as the independent term and the corrected left thalamus volumes as to the dependent variable, are presented.

A similar trait was observed in the right thalamus, not surviving to multiple comparisons. Importantly, no other subcortical region has shown significance for cortisol measurements.

## 4 Discussion

In the present study, we explore how psychosocial stress correlates with subcortical brain morphometry, using a cohort of healthy adults. Results show a positive association between perceived stress and subcortical regions, in particular, the right amygdala, linked to emotional processing.

Exposure to stress is part of life. Importantly, each subject perceives stress differently. Assuming the concept of stress perception as a continuum (Selye, 1956), we describe here a positive association between stress perception and the volume of subcortical brain regions implicated in emotional processing. The predominant effect herein observed was in the right amygdala, with a similar tendency on the left hemisphere not surviving to multiple comparisons in FreeSurfer analysis. Importantly, besides the well-stated involvement of the amygdala in emotional processing, and its role mediating stress-responses (LeDoux, 2000), studies have shown that amygdala neuroplasticity is associated with the subject's psychological state (Taren et al., 2013). Indeed, the longitudinal VBM study of Hölzel et al. in a healthy, however, stressed cohort, revealed that after an effective stress-reduction intervention, the decrease in PSS levels matched with a reduction in the right basolateral amygdala gray matter density (Hölzel et al., 2010).

Interestingly, and despite a similar trend, the present association seems to be stronger on the right hemisphere. Highlighting the right dominance on affective and emotional processes (namely on the stress response modulation), and its contrast to the left prominence on language and motor functions (Cerqueira et al., 2008), studies have shown evidences of specific functional asymmetries. Particularly in the amygdala, variations in the affective processing are point out as a rationale for the divergences observed (Hölzel et al., 2010; Lanteaume et al., 2007; Markowitsch, 1999). Here, the fast initial, and possibly automatic, response of the right amygdala to stimuli contrasts to a further discriminative evaluation of the stressor by the left amygdala (Hölzel et al., 2010). Indeed, a clinical study revealed that right amygdala stimulation triggered negative emotions and left amygdala stimulation induced both pleasant and unpleasant emotions (Lanteaume et al., 2007). Interestingly, and in contrast, the recent study of Wu et al. has also shown a significant interaction effect of stress by age on a cluster extending to the left amygdala (Wu et al., 2020); indeed, post-hoc comparisons revealed a positive association with left amygdala volumes in adolescents, with middle-aged adults presenting a negative correlation with PSS levels, which may justify the weaker effect observed on the left hemisphere.

When focusing only on the hippocampus, our results contrast with the usual atrophy observed in stressed subjects (Cameron and Schoenfeld, 2018; Gilbertson et al., 2002; Zimmerman et al., 2016). Yet, it is important to highlight the fact that there is a functional and a structural connectivity segregation between the anterior vs posterior hippocampus (Sousa, 2016) and also that previous studies assessing the impact of stress, namely in rodents have shown a clear volumetric differential response with atrophy in dorsal hippocampus and hypertrophy in the ventral component (more related to the anterior hippocampus in humans) (Pinto et al., 2015). Interestingly, our findings also show distinct profiles across hemispheres. On the right hemisphere, volumetric changes are noted on the anterior hippocampus, a region known for its role in mediating emotional and affective processes (Strange et al., 2014). This is a novel finding, as in a similar study, Li et al. have shown a positive association not with the anterior hippocampus but, between the anterior parahippocampal gyrus and PSS (Li et al., 2014). Actually, this slight disparity upon results should be carefully interpreted, taking particular attention to the methodological differences between studies. Most importantly, a distinguished preprocessing pipeline and morphometry analysis (using SPM8; https://www.fil.ion.ucl.ac.uk/spm/software/spm8/), represent variables that if ignored could lead to a misinterpretation of results (Popescu et al., 2016). In fact, Rajagopalan and Pioro have reported that analyses in distinct software (FSL, SPM, and FreeSurfer) lead to disparate VBM results, which enhances the importance of performing complementary analysis in the same study (Rajagopalan and Pioro, 2015). Indeed, the present study illustrates this fact, as the findings on the anterior hippocampus on the right, and posterior hippocampus on the left, are only observed with FSL-VBM analysis, but not with FreeSurfer.

A final note to highlight the findings in the putamen and nucleus accumbens in our FSL-VBM, but not FreeSurfer, analysis. The putamen is involved in distinct brain functions - such as learning, cognitive functioning, or reward (Ghandili and Munakomi, 2020) - previous reports from our group have already shown hypertrophy in sensorimotor corticostriatal circuits, which is accompanied by a shift to habit-behavior strategies (Soares et al., 2012). The nucleus accumbens gray matter increases have been linked to anxiety (Kühn et al., 2011; Lee et al., 2020), being also identified as a biomarker for treatment-responders (Burkhouse et al., 2020). Nonetheless, the residual effect observed in all these regions, that were not confirmed in our FreeSurfer analysis, preclude any conclusion on relevant associations between the volumes of these brain regions and perceived stress scores.

A considerable sex difference in sample size is a limitation in our study. However, we have tackled this limitation by doing corrections for sex through the use of a covariate in all the analyses. Another question that can be pointed out as a limitation in our study is the predominant use of PSS10, a subjective instrument that measures the individual perception of stress and not an objective quantification of stress itself. In fact, studies have struggled in showing consistent associations between cortisol and psychosocial stressors (Chida and Steptoe, 2009; Halford et al., 2012), contrasting to the well-established PSS instrument (Fliege et al., 2005; Soares et al., 2012; Trigo et al., 2010). Therefore, and bearing in mind that distress only exists when recognized by the subject (Goldstein and Kopin, 2007), we consider that this metric is of great physiological (and even clinical) relevance. Indeed, the current data confirms that the way subjects integrate stress is associated with brain morphometry, regardless of the true amount of exposure or kind of stressor. On the other hand, the additional analysis conducted on a subgroup of our participants endorses the fact that endocrine measures, namely cortisol, do not associate equally with the volume of subcortical brain regions. Our results show a negative association between cortisol and left thalamus volumes, which contrast to previous observations in an older cohort (Lau et al., 2017), and emphasizes the volumetric sex differences that have been previously reported by others (Koolschijn and Crone, 2013; Menzler et al., 2011). Indeed, using a single measurement of salivary cortisol, we are addressing an acute endocrine response that contrasts with the chronic, one-month evaluation of psychological stress measured by with PSS10 questionnaire. However, in future work, we do not exclude the possibility to combine psychological questionnaires with physiological measures, to further explore the relationship between perceived and objective stress measures, as suggested by Lee (2012). In fact, Lazarides et al. argue that the failure in demonstrating this link arises from limitations in measurements approaches and, most importantly, on the reliance of between-subject comparisons, rather than within-subject associations (Lazarides et al., 2020).

In conclusion, herein we explored the association of psychosocial stress in subcortical brain regions volumes, using a non-pathological population. We performed a volumetric regression analysis where perceived stress scores were used as a variable of interest and we demonstrate that increased levels of perceived stress positively associate with the right amygdala and anterior hippocampal volumes.

## CRediT authorship contribution statement

**Inês Caetano:** Conceptualization, Formal analysis, Validation, Writing – original draft, Visualization, contributed to the study design, Formal analysis, interpreted the results, discussed the manuscript, wrote the first draft of the manuscript, performed data presentation. **Liliana Amorim:** Investigation, Data curation, performed participant's recruitment, neuropsychological assessment, Formal analysis, manuscript discussion. **José Miguel Soares:** Investigation, Data curation, performed participant's recruitment, MRI acquisitions. **Sónia Ferreira:** Formal analysis, Formal analysis, interpretation of results. **Ana Coelho:** Software, Visualization, Formal analysis, presentation of results. **Joana Reis:** Formal analysis, Formal analysis. **Nadine Correia Santos:** Funding acquisition, provided the resources for study development. **Pedro Silva Moreira:** Methodology, Formal analysis, Methodology, Formal analysis. **Paulo Marques:** Investigation, performed the MRI acquisitions. **Ricardo Magalhães:** Investigation, Visualization, performed the MRI acquisitions, contributed to the presentation of results. **Madalena Esteves:** Software, Formal analysis, Validation, Formal analysis. **Maria Picó-Pérez:** Software, Formal analysis, Validation, performed the data pre-processing, Formal analysis, manuscript discussion. **Nuno Sousa:** Conceptualization, Validation, Writing – review & editing, Supervision, Funding acquisition, conceived the study design, provided the resources for study development, reviewed the acquired data, interpreted the results, performed manuscript discussion, reviewed the final manuscript.

## Declaration of competing interest

The authors declared that they had no conflicts of interest concerning their authorship or the publication of this article.

## Appendix A Supplementary data

Multimedia component 5 Multimedia component 5

figs1 figs1
