# Supplementary material for: Amygdala size varies with stress perception
Source: Neurobiol Stress. 2021 May 1;14:100334. doi: 10.1016/j.ynstr.2021.100334 (PMC8114169; doi:10.1016/j.ynstr.2021.100334)
Supplement: Multimedia component 1 [file mmc1.docx]

**Fig. A.1. Representation of the multilinear model resulted from left thalamus regression with cortisol.** A statistically significant association between left thalamus volumes and both cortisol (negative) and sex (positive) independent terms was observed, even after correction for multiple comparisons. Note that the direction of the sex association is only related to the way sex covariate was codified (females as 0 and males as 1). Therefore, the positive association between left thalamus volumes and sex indicates that being a male positively contribute to having a bigger volume of left thalamus, and, in contrast, being a female contribute to having smaller left thalamus volume. On left, representation of the *10_L Thalamus* cluster from FreeSurfer subcortical regions labeling. In the middle, graphical representation of the model with cortisol measurements as the independent variable and left thalamus volumes, corrected for age and sex covariates, as dependent variable; the equation represents the correlation between cortisol measurements and corrected thalamus volumes, where statistical significance is observed. On the right, a graphical representation of the model with sex as the independent factor and left thalamus volumes, corrected for age and cortisol measurements, as the dependent variable; herein an independent-sample t-test indicates that left thalamus volumes are significantly higher for males than for females (on graph, mean volumes ± standard deviation for each level are presented).

Brain volumes were computed using FreeSurfer subcortical output and corrected for individual GM. To avoid a large number of decimal digits, corrected volumes were multiplied by 100. Multilinear regression models with ROI volumes as dependent variable and cortisol measurements, age, and sex as independent terms were established. The models were computed using the *regstats* function in *MATLAB* and the Bonferroni-Holm correction for 14 multiple comparisons was used to calculate the corrected *p*-values. Statistical significance was established for *α* = 0.05.
