# Supplementary material for: Amygdala size varies with stress perception
Source: Neurobiol Stress. 2021 May 1;14:100334. doi: 10.1016/j.ynstr.2021.100334 (PMC8114169; doi:10.1016/j.ynstr.2021.100334)
Supplement: Multimedia component 2 [file mmc2.docx]

**Table A.1. Descriptive statistics of subcortical volumes obtain through FreeSurfer.** Brain volumes were computed using FreeSurfer subcortical output (*aseg.stats*), and corrected for individual GM to best replicate the FSL-VBM pipeline. To avoid using further decimal digits, corrected volumes were multiplied by 100.

|  | | **Volumes (mm^3^)** | |  | **Volumes/GM** | |
| --- | --- | --- | --- | --- | --- | --- |
| Subcortical ROI | | Mean | Standard Deviation |  | Mean | Standard Deviation |
|  | 10_L Thalamus-Proper | 7759 | 904 |  | 1.173 | 0.073 |
|  | 49_R Thalamus-Proper | 7413 | 797 |  | 1.121 | 0.061 |
|  | 11_L Caudate | 3725 | 490 |  | 0.564 | 0.059 |
|  | 50_R Caudate | 3842 | 506 |  | 0.582 | 0.057 |
|  | 12_L Putamen | 5015 | 501 |  | 0.760 | 0.061 |
|  | 51_R Putamen | 5017 | 527 |  | 0.760 | 0.061 |
|  | 13_L Pallidum | 1985 | 236 |  | 0.301 | 0.028 |
|  | 52_R Pallidum | 1924 | 241 |  | 0.291 | 0.027 |
|  | 17_L Hippocampus | 3978 | 475 |  | 0.602 | 0.046 |
|  | 53_R Hippocampus | 4048 | 466 |  | 0.613 | 0.048 |
|  | 18_L Amygdala | 1404 | 208 |  | 0.212 | 0.023 |
|  | 54_R Amygdala | 1497 | 258 |  | 0.226 | 0.029 |
|  | 26_L Accumbens-area | 625 | 91 |  | 0.095 | 0.014 |
|  | 58_R Accumbens-area | 572 | 82 |  | 0.087 | 0.012 |
| General Volumes | |  |  |  |  |  |
|  | eTIV | 1.59x10^6^ | 1.51x10^5^ |  |  |  |
|  | GM | 6.61x10^5^ | 5.50x10^4^ |  |  |  |
|  | WM | 4.68x10^5^ | 5.89x10^4^ |  |  |  |
|  |  |  |  |  |  |  |
| *VBM. Voxel-based-morphometry; ROI. Region-of-interest; eTIV. Estimated Intracranial Volume; GM. Gray matter; WM. White matter; R. Right; L. Left.* | | | | | | |
