# Supplementary material for: Amygdala size varies with stress perception
Source: Neurobiol Stress. 2021 May 1;14:100334. doi: 10.1016/j.ynstr.2021.100334 (PMC8114169; doi:10.1016/j.ynstr.2021.100334)
Supplement: Multimedia component 3 [file mmc3.docx]

**Table A.2. Results from FreeSurfer subcortical volumetric regression with PSS.** A statistically significant positive association between PSS and right amygdala volume is observed, even when correcting for multiple comparisons. Positive associations between PSS and right pallidum, right hippocampus, left amygdala and right accumbens-area did not survive multiple comparison correction.

Brain volumes were computed using FreeSurfer subcortical output (*aseg.stats*) and corrected for individual GM to best replicate the FSL-VBM regression analysis. To avoid using further decimal digits, corrected volumes were multiplied by 100. Multilinear regression models with ROI volumes as dependent variables and PSS, age, and sex as independent variables were established. The models were computed using the function *regstats* in *MATLAB* and the Bonferroni-Holm correction for 14 multiple comparisons was used to calculate the corrected *p*-values. For easy interpretation, *p*- and corrected *p*-values statistically significant are presented in bold, as the respective ROI description and effect size when significance is observed in PSS independent term. Statistical significance was established for α = 0.05.

| **ROI** | |  | | **MULTILINEAR REGRESSION** | | | | | | | | | | | |  |
| --- | --- | --- | --- | --- | --- | --- | --- | --- | --- | --- | --- | --- | --- | --- | --- | --- |
|  |  | **PSS** | | | |  | **Age** | | |  | **Sex** | | |  | **Model Effect size** | |
|  |  | ***p*-value** | **Corrected *p*-value** | | **Slope (β)** |  | ***p*-value** | **Corrected *p*-value** | **Slope (β)** |  | ***p*-value** | **Corrected *p*-value** | **Slope (β)** |  | **R^2^** | **Adjusted R^2^** |
| Subcortical | |  |  | |  |  |  |  |  |  |  |  |  |  |  |  |
|  | 10_L Thalamus-Proper | 0.181 | 1.432 | | 0.0020 |  | 0.728 | 2.006 | 0.0020 |  | **0.049** | 0.680 | 0.0230 |  | 0.113 | 0.054 |
|  | 49_R Thalamus-Proper | 0.630 | 2.520 | | 0.0006 |  | 0.470 | 2.822 | 0.0035 |  | 0.120 | 1.558 | 0.0152 |  | 0.071 | 0.010 |
|  | 11_L Caudate | 0.965 | 1.604 | | 0.0001 |  | 0.549 | 2.822 | 0.0029 |  | 0.888 | 1.369 | 0.0014 |  | 0.008 | 0.056 |
|  | 50_R Caudate | 0.283 | 1.697 | | 0.0012 |  | 0.641 | 2.743 | 0.0020 |  | 0.518 | 4.207 | 0.0056 |  | 0.032 | 0.033 |
|  | 12_L Putamen | 0.381 | 1.907 | | 0.0011 |  | 0.174 | 1.389 | 0.0068 |  | 0.590 | 3.686 | 0.0052 |  | 0.064 | 0.003 |
|  | 51_R Putamen | 0.756 | 2.520 | | 0.0004 |  | 0.120 | 1.436 | 0.0078 |  | 0.666 | 3.538 | 0.0043 |  | 0.063 | 0.001 |
|  | 13_L Pallidum | 0.802 | 2.267 | | 0.0001 |  | 0.105 | 1.366 | 0.0036 |  | 0.467 | 4.386 | 0.0031 |  | 0.075 | 0.014 |
|  | **52_R Pallidum** | **0.025** | 0.271 | | 0.0012 |  | 0.143 | 1.417 | 0.0030 |  | 0.168 | 2.021 | 0.0056 |  | **0.180** | **0.126** |
|  | 17_L Hippocampus | 0.093 | 0.838 | | 0.0015 |  | 0.933 | 1.457 | 0.0003 |  | 0.679 | 2.706 | 0.0029 |  | 0.071 | 0.009 |
|  | **53_R Hippocampus** | **0.021** | 0.248 | | 0.0021 |  | 0.126 | 1.436 | 0.0053 |  | 0.409 | 4.498 | 0.0057 |  | **0.184** | **0.130** |
|  | **18_L Amygdala** | **0.013** | 0.163 | | 0.0011 |  | 0.343 | 2.401 | 0.0017 |  | 0.684 | 2.038 | 0.0014 |  | **0.156** | **0.100** |
|  | **54_R Amygdala** | **< 0.001** | **< 0.001** | | 0.0022 |  | 0.142 | 1.417 | 0.0027 |  | 0.527 | 4.140 | 0.0023 |  | **0.382** | **0.341** |
|  | 26_L Accumbens-area | 0.179 | 1.432 | | 0.0003 |  | **0.026** | 0.363 | 0.0023 |  | 0.439 | 4.498 | 0.0015 |  | 0.130 | 0.072 |
|  | **58_R Accumbens-area** | **0.033** | 0.326 | | 0.0004 |  | 0.669 | 2.565 | 0.0003 |  | 0.676 | 3.331 | 0.0007 |  | **0.111** | **0.051** |
| *VBM. Voxel-based-morphometry; ROI. Region-of-interest; R. Right; L. Left.* | | | | | | | | | | | | | | | | |
