# Supplementary material for: Amygdala size varies with stress perception
Source: Neurobiol Stress. 2021 May 1;14:100334. doi: 10.1016/j.ynstr.2021.100334 (PMC8114169; doi:10.1016/j.ynstr.2021.100334)
Supplement: Multimedia component 4 [file mmc4.docx]

**Table A.3. Results from FreeSurfer hippocampal subfields association with PSS.** A statistically significant positive association between PSS and right anterior hippocampus is observed. No statistically significant association is observed between the right posterior hippocampus and PSS scores.

After the FreeSurfer segmentation (*aseg.stats*), the hippocampal segmentation was made (*hipposubfields.rh.T1.v21.stats*) and the subfields grouped into anterior hippocampus (head) and posterior hippocampus (body and tail). Individual correction for GM volumes was made, and corrected volumes were multiplied by 100 to avoid using further decimal digits. Multilinear regression models with ROI volumes as dependent variables and PSS, age, and sex as independent variables were established. The models were computed using the function *regstats* in *MATLAB* and the Bonferroni-Holm correction for 2 multiple comparisons was used to calculate the corrected *p*-values. For easy interpretation, *p*- and corrected *p*-values statistically significant are presented in bold, as the respective region and effect size, when significance is observed in PSS independent term. Statistical significance was established for α = 0.05.

| **ROI** | |  | | **MULTILINEAR REGRESSION** | | | | | | | | | | | |  |
| --- | --- | --- | --- | --- | --- | --- | --- | --- | --- | --- | --- | --- | --- | --- | --- | --- |
|  |  | **PSS** | | | |  | **Age** | | |  | **Sex** | | |  | **Model Effect size** | |
|  |  | ***p*-value** | **Corrected *p*-value** | | **Slope (β)** |  | ***p*-value** | **Corrected *p*-value** | **Slope (β)** |  | ***p*-value** | **Corrected *p*-value** | **Slope (β)** |  | **R^2^** | **Adjusted R^2^** |
| R Hippocampus | |  |  | |  |  |  |  |  |  |  |  |  |  |  |  |
|  | **Anterior** | **0.012** | **0.023** | | 0.0011 |  | 0.075 | 0.075 | 0.0031 |  | 0.379 | 0.757 | -0.0060 |  | **0.219** | **0.166** |
|  | Posterior | 0.287 | 0.287 | | 0.0004 |  | **0.025** | **0.049** | 0.0038 |  | 0.422 | 0.757 | -0.0052 |  | 0.147 | 0.090 |
| *VBM. Voxel-based-morphometry; ROI. Region-of-interest; R. Right; L. Left.* | | | | | | | | | | | | | | | | |
