# Supplementary material for: Amygdala size varies with stress perception
Source: Neurobiol Stress. 2021 May 1;14:100334. doi: 10.1016/j.ynstr.2021.100334 (PMC8114169; doi:10.1016/j.ynstr.2021.100334)
Supplement: Multimedia component 5 [file mmc5.docx]

**Table A.4. Models resulted from the volumetric regression with cortisol measurements.** Multilinear regression models with ROI volumes as dependent variable and cortisol measurements, age, and sex as independent variables were established. A statistically significant negative association between cortisol and left thalamus is observed, as well as a significant positive association between sex and the left thalamus volumes. Note that the direction of the sex association is only related to the way sex covariate was codified (females as 0 and males as 1). Therefore, the positive association between left thalamus volumes and sex indicates that being a male positively contribute for having a bigger volume of left thalamus, and, in contrast, being a female contribute for having smaller left thalamus volume. A similar trait in the right thalamus did not survive to multiple comparisons, such as the associations between age and bilateral putamen, and between sex and right pallidum. The negative value of the adjusted *R^2^* of left caudate, bilateral hippocampus, and bilateral amygdala indicates that the multilinear models are not appropriate for the data.

Brain volumes were computed using FreeSurfer subcortical output (*aseg.stats*) and corrected for individual GM and multiplied by 100. The models were computed using the function *regstats* in *MATLAB* and the Bonferroni-Holm correction for 14 multiple comparisons was used to calculate the corrected *p*-values. For easy interpretation, *p*- and corrected *p*-values statistically significant are presented in bold, as the respective ROI description and model’s effect sizes when significance is observed in cortisol independent term. Statistical significance was established for α = 0.05.

| **ROI** | |  | | **MULTILINEAR REGRESSION** | | | | | | | | | | | |  |
| --- | --- | --- | --- | --- | --- | --- | --- | --- | --- | --- | --- | --- | --- | --- | --- | --- |
|  |  | **Cortisol** | | | |  | **Age** | | |  | **Sex** | | |  | **Model Effect size** | |
|  |  | ***p*-value** | **Corrected *p*-value** | | **Slope (β)** |  | ***p*-value** | **Corrected *p*-value** | **Slope (β)** |  | ***p*-value** | **Corrected *p*-value** | **Slope (β)** |  | **R^2^** | **Adjusted R^2^ *** |
| Subcortical | |  |  | |  |  |  |  |  |  |  |  |  |  |  |  |
|  | **10_L Thalamus-Proper** | **0.0016** | **0.0221** | | -0.1961 |  | 0.4494 | 2.6963 | -0.0065 |  | **< 0.0001** | **0.0005** | 0.1347 |  | **0.599** | **0.542** |
|  | **49_R Thalamus-Proper** | **0.0065** | 0.0844 | | -0.1770 |  | 0.9404 | 1.1632 | -0.0007 |  | **0.0102** | 0.1320 | 0.0790 |  | 0.388 | 0.301 |
|  | 11_L Caudate | 0.9227 | 1.8367 | | -0.0068 |  | 0.5740 | 2.0783 | 0.0062 |  | 0.8614 | 3.3306 | -0.0058 |  | 0.017 | -0.123 |
|  | 50_R Caudate | 0.1672 | 1.8395 | | -0.0777 |  | 0.5196 | 2.5157 | 0.0055 |  | 0.5066 | 2.5330 | -0.0176 |  | 0.156 | 0.030 |
|  | 12_L Putamen | 0.1814 | 1.7403 | | -0.0857 |  | **0.0488** | 0.6346 | 0.0204 |  | 0.2957 | 2.0699 | 0.0318 |  | 0.307 | 0.208 |
|  | 51_R Putamen | 0.3259 | 1.9552 | | -0.0648 |  | **0.0246** | 0.3445 | 0.0245 |  | 0.3449 | 2.0699 | 0.0297 |  | 0.326 | 0.229 |
|  | 13_L Pallidum | 0.2092 | 1.5046 | | -0.0298 |  | 0.2419 | 2.0660 | 0.0044 |  | 0.1220 | 1.2197 | 0.0177 |  | 0.242 | 0.134 |
|  | 52_R Pallidum | 0.1740 | 1.8395 | | -0.0363 |  | 0.5816 | 1.7220 | 0.0023 |  | **0.0172** | 0.2063 | 0.0319 |  | 0.306 | 0.207 |
|  | 17_L Hippocampus | 0.8942 | 4.2712 | | -0.0068 |  | 0.2296 | 2.0660 | 0.0096 |  | 0.8915 | 2.5843 | 0.0034 |  | 0.081 | -0.056 |
|  | 53_R Hippocampus | 0.9183 | 2.7069 | | -0.0056 |  | 0.1945 | 1.9449 | 0.0112 |  | 0.9739 | 1.7831 | -0.0009 |  | 0.088 | -0.049 |
|  | 18_L Amygdala | 0.8542 | 4.2712 | | -0.0051 |  | 0.1226 | 1.4708 | 0.0070 |  | 0.8327 | 3.3306 | -0.0028 |  | 0.119 | -0.006 |
|  | 54_R Amygdala | 0.9023 | 3.5767 | | -0.0043 |  | 0.5031 | 2.6963 | 0.0037 |  | 0.2502 | 2.0013 | 0.0194 |  | 0.119 | -0.007 |
|  | 26_L Accumbens-area | 0.0860 | 1.0323 | | -0.0196 |  | 0.3043 | 2.1299 | 0.0018 |  | 0.1076 | 1.1835 | 0.0087 |  | 0.264 | 0.159 |
|  | 58_R Accumbens-area | 0.1881 | 1.6330 | | -0.0144 |  | 0.1550 | 1.7053 | -0.0025 |  | 0.1786 | 1.6071 | 0.0070 |  | 0.152 | 0.031 |
| *VBM. Voxel-based-morphometry; ROI. Region-of-interest; R. Right; L. Left; * A negative R^2^ statistic indicates that the model is not appropriate for the data.* | | | | | | | | | | | | | | | | |
